# The Yang-Tonifying Herbal Medicine *Cynomorium songaricum* Extends Lifespan and Delays Aging in *Drosophila*

**DOI:** 10.1155/2012/735481

**Published:** 2012-07-15

**Authors:** Hsin-Ping Liu, Rong-Fu Chang, Yih-Shyuan Wu, Wei-Yong Lin, Fuu-Jen Tsai

**Affiliations:** ^1^Graduate Institute of Acupuncture Science, China Medical University, Taichung 40402, Taiwan; ^2^Department of Biotechnology, Asia University, Taichung 41354, Taiwan; ^3^Graduate Institute of Chinese Medicine, China Medical University, Taichung 40402, Taiwan; ^4^Graduate Institute of Integrated Medicine, China Medical University, Taichung 40402, Taiwan; ^5^Department of Medical Research, China Medical University Hospital, Taichung 40402, Taiwan

## Abstract

Aging is highly correlated with the progressive loss of physiological function, including cognitive behavior and reproductive capacity, as well as an increased susceptibility to diseases; therefore, slowing age-related degeneration could greatly contribute to human health. *Cynomorium songaricum* Rupr. (CS) is traditionally used to improve sexual function and treat kidney dysfunction in traditional Chinese medicine, although little is known about whether CS has effects on longevity. Here, we show that CS supplementation in the diet extends both the mean and maximum lifespan of adult female flies. The increase in lifespan with CS was correlated with higher resistance to oxidative stress and starvation and lower lipid hydroperoxides (LPO) levels. Additionally, the lifespan extension was accompanied by beneficial effects, such as improved mating readiness, increased fecundity, and suppression of age-related learning impairment in aged flies. These findings demonstrate the important antiaging effects of CS and indicate the potential applicability of dietary intervention with CS to enhance health and prevent multiple age-related diseases.

## 1. Introduction

Age is the most important risk factor for the progressive loss of physiological function, increased susceptibility to diseases and mortality due to the gradual accumulation of damage at the cellular, molecular, and tissue levels [1]. Because the aging process is controlled by complicated mechanisms and driven by the broad influences of genetic and environmental factors, research has often shown that age-related pathology, such as a shorter lifespan, cancer, and neurodegenerative diseases, could be delayed through genetic or pharmacological manipulation [2–4]. A main focus in study of aging is energy homeostasis and metabolic processes. In systems ranging from unicellular yeast to worms, flies, rodents, and primates, a decrease in nutrient uptake by organisms during dietary restriction (DR) has been demonstrated to effectively extend the lifespan and exert beneficial effects in delaying the progression of age-related diseases [5].

Accumulating evidence suggests that nutritional supplementation also increases lifespan and delays age-related syndromes. For example, catechin extracts, including epigallocatechin gallate (EGCG), from green tea prolong the lifespan and increase the antioxidative activity of *Drosophila* and *Caenorhabditis elegans* [6, 7]. Curcumin, an active ingredient of the spice *Curcuma longa* (turmeric), has demonstrated protection against oxidative stress and aging in several animals [8, 9]. Resveratrol, a main component of red wine, extends lifespan, increases oxidative resistance and prevents diet-induced metabolic syndrome in several model organisms, including flies, yeast, nematodes, mice, and a short-lived fish [10]. This small polyphenol molecule also participates in maintaining the youth of endothelial progenitor cells by modulating telomerase activity [11]. Approaches to the DR and resveratrol-dependent molecular mechanisms, sirtuin, and insulin signaling pathways are essential for these prolongevity effects [12, 13].

Given that natural compounds can decrease mortality, we considered an alternative strategy to identify traditional Chinese medicines that have prolongevity effects. In an unbiased screening test for a lower incidence of mortality in the *Drosophila melanogaster*, we identified a herbal medicine with a potential effect. *Cynomorium songaricum *Rupr. (CS), also known by its Chinese name, *Suoyang*, is a parasitic plant that grows in northwest China. Extracted from its stem is a widely used Chinese herbal remedy for treating sexual dysfunction, infertility, deficient kidney function, and lumbar weakness, as well as for facilitating catharsis, as described in ancient Chinese medical books. Chemical components identified in CS extracts include organic acids, triterpenes, flavonoids, polysaccharide, and steroidal compounds [14–16]. A recent report described the potent effect of CS extracts on promoting spermatogenesis in rat testes as mediated through glial cell-derived neurotrophic factor (GDNF) stimulation, supporting the traditional utilization of CS for male sexual dysfunction [17]. CS extracts might also have estrogenic activities for female reproductive tissues that would alleviate menopausal symptoms and play a regulatory role on stimulating osteoblast proliferation to prevent further bone loss in osteoporosis [18, 19]. Other biomedical research has reported that triterpenes from CS extracts have protease inhibitory activity against HIV-1 and HCV proliferation [20, 21]. Yu et al. showed that CS supplementation can increase the activity of reactive oxygen species (ROS) scavenging enzyme and enhance exercise performance in rats [22]. Despite its various documented beneficial effects, the correlation between CS and its effects on oxidative stress resistance, antiaging, and cognitive function has not been elucidated. To investigate the mechanisms underlying the role of CS in antisenescence, we adopted a pharmacological approach using *Drosophila*, which is an ideal organism for *in vivo* aging studies. Our data show significant extension of lifespan, improved resistance to environmental stress, and suppressed age-related syndromes, suggesting the possible clinical utility of CS in slowing the aging process.

## 2. Materials and Methods

### 2.1. Fly Stock Maintenance and Lifespan Measurement

The wild-type fly line used in this study was of the *Canton S* background and maintained in cornmeal standard media at 25°C under a 12 h light-dark cycle. Emerging adult flies were collected within 24 h and separated by sex. From early adulthood, flies were supplemented with CS as a concentrated herbal medicine purchased from Ko Da Pharmaceutical Co., Ltd. (Taoyuan, Taiwan) under the good manufacturing practice (GMP) criteria. All experimental procedures were performed at 29°C with 50–60% relative humidity. CS powder was dissolved in H_2_O and added to cornmeal media at 10, 20, and 30 mg/mL concentrations, which were proportional to the effective daily dosage for human oral administration. In a lifespan analysis, twenty to thirty flies were raised in a food vial with or without CS; at least four vials were prepared for each treatment. Food vials were replaced every 2 to 3 days, and dead flies were counted at that time. At least three replicates were conducted per trial.

### 2.2. Stress Resistance Assays

Female flies were collected in a vial and pretreated with various doses of CS for 20 days at 29°C. Before testing, the flies were starved for 6 h. For oxidative stress assays, flies were transferred to food-free vials containing a filter paper saturated with 10 mM paraquat (Sigma, St. Louis, MO) diluted in a 5% glucose solution. Paraquat generates superoxide anions (O_2_^•−^) that cause oxidative damage to animals, and we thus measured the resistance of the flies to this oxidative stress. Similarly, flies were transferred to vials containing a filter paper with hydrogen peroxide (H_2_O_2_) (Sigma, St. Louis, MO) in a 5% glucose solution to gauge the flies' resistance against H_2_O_2_-induced hydroxyl radical (OH^•−^) stress. We also kept flies in vials with wet papers only and evaluated their stamina against starvation-induced oxidative stress. Flies were scored for mortality at least three times daily until all were dead. Every 24 h, flies were transferred to new vials with freshly prepared solutions. At least three vials per treatment were performed, and all experiments were conducted in triplicate.

### 2.3. Antioxidant Enzyme Activity

Changes in antioxidant activity after CS treatment were evaluated by measuring catalase and superoxide dismutase (SOD) activity using catalase and SOD assay kits (Cayman Chemical, MI). Briefly, female flies were reared on a control or experimental diet containing different doses of CS at 29°C for 20 days. For each enzyme activity assay, flies in each group were collected and homogenized in cold buffer. Supernatants were collected after centrifugation and assayed for catalase and CuZn-SOD enzyme activity according to the kit's protocol.

### 2.4. Lipid Hydroperoxides (LPO) Assay

LPO was evaluated by quantifying the malondialdehyde (MDA) level using a thiobarbaturic acid (TBA) (Sigma, St. Louis, MO) method [23]. Briefly, female flies pretreated with various doses of CS for 20 days at 29°C were collected and homogenized for LPO analysis. Hydroperoxides in the sample were extracted with methanol and reacted with TCA-TBA-HCl solution (15% trichloroacetic acid (TCA), 0.375% TBA in 0.2 N HCl). The mixture was heated in a boiling water bath for 30 min. The absorbance of each sample was determined at 532 nm, and MDA content was quantified using 1,1,3,3-tetramethoxypropane (Sigma, St. Louis, MO) as a standard.

### 2.5. Courtship Assays

Virgin females were collected and treated with different doses of CS for 10 or 20 days as experimental groups. The day of eclosion was designated as day 1 of adult life. To measure courtship behavior, one experimental female was paired with a virgin male, which was 9-day-old and had not received CS supplementation, in a plastic chamber. The experiment was conducted at Zeitgeber time (ZT) 3 to ZT6, which is the time that females show higher mating activities [24]. ZT0 and ZT12 indicate the times that lights turn on and off, respectively, in a 12 h light-dark cycle at 25°C. The time when each couple encountered and completed copulation was recorded with a camera and determined with an accuracy of 1 sec. The copulation duration for each couple was assigned by the times at which couples initiated and terminated copulation, and the copulation latency was defined as the times between which the couples were placed in the chamber and started to copulate.

### 2.6. Fecundity Assessment

Virgin females fed varying doses of CS for either 10 or 20 days served as experimental groups. Ten experimental females were mated with an equal number of virgin males, which were 9-day-old and had not received CS, for 24 h in a vial. The mated flies were then transferred to a new vial for egg laying for 24 h, and the total eggs laid in the vials were counted manually. An index of fecundity was calculated as the mean daily egg production per female. At least five vials per treatment were performed (*n* = 50–60).

### 2.7. Olfactory Associative Learning

Female flies were collected after eclosion and pretreated with 20 mg/mL CS for 20 days at 29°C. One training session was performed according to a previous study [25]. Briefly, approximately 100 flies were exposed sequentially to two odors, either 3-octanol (OCT) or 4-methylcyclohexanol (MCH). Flies exposed to the first odor (conditioned stimulus, CS^+^) were paired with 60 V electric shocks and then received a second odor (CS^*‒*^) without shocks. Learning ability was determined immediately after training. To perform the test, the trained flies were trapped into the choice point of a T-maze in which they were exposed simultaneously to OCT and MCH. A performance index (PI) was calculated to represent the conditioned odor avoidance. A PI of zero indicated a 50 : 50 distribution, and a PI of 100 showed a 0 : 100 distribution away from the CS^+^ odor.

### 2.8. Statistical Analysis

All experiments were conducted with at least three independent replicates, and statistical analyses were performed with SigmaStat v3.5 software (Systat Software, Inc., IL). Data for lifespan extension and oxidative stress challenges represented experiments performed with a total number of flies ranging from 250–350, and the log-rank test was assessed for difference analysis. The copulation latency was not distributed normally, thus we used nonparamertric Mann-Whitney *U*-test. Other experimental comparisons were made by performing a Student's *t*-test compared with the control groups. Data are represented as the mean ± SEM, and a *P* value of <0.05 was considered significant.

## 3. Results

### 3.1. CS Supplementation Increases Lifespan in Flies

**Figure 1:**
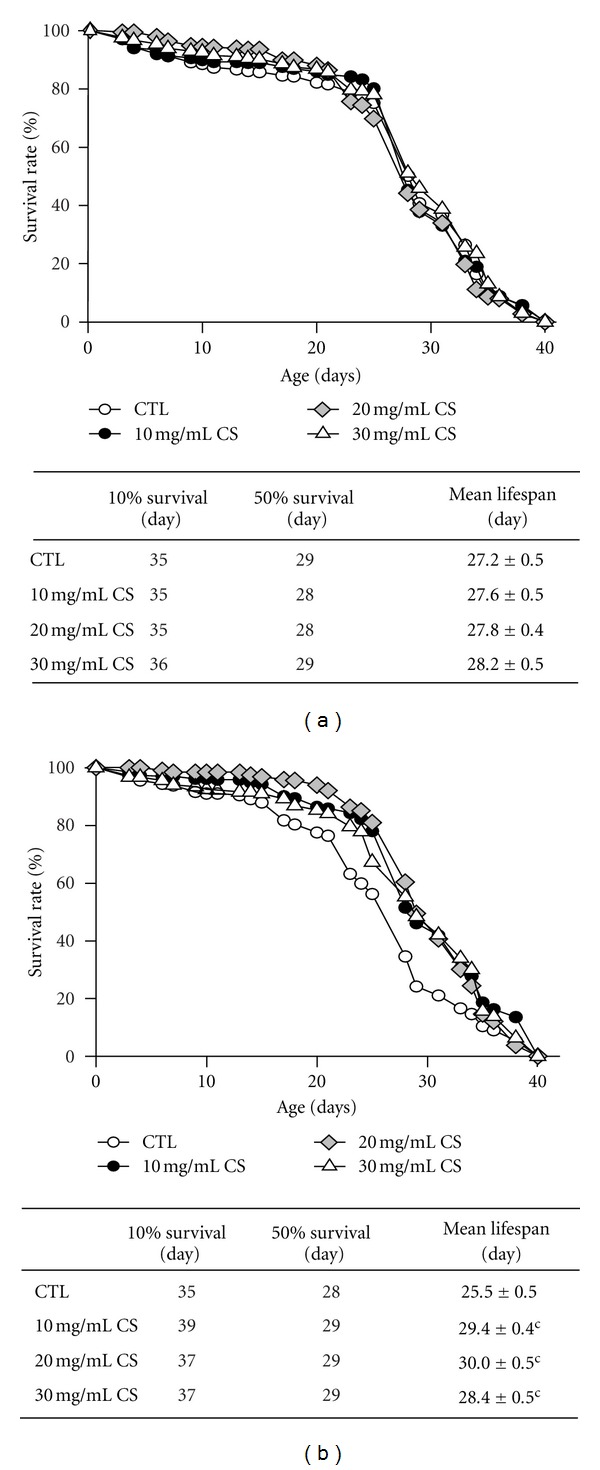
Effects of CS on lifespan. Flies fed the control diet (CTL, open circle) and experimental diets supplemented with 10 (closed circle), 20 (open diamond), and 30 mg/mL (open triangle) CS at 29°C. Data represent the total lifespan of tested (a) male and (b) female flies. Data are shown as the mean ± SEM and significantly different from the controls for each treatment at ^c^ *P* < 0.001.

To examine whether the application of CS can extend the fly lifespan, flies were supplemented with 10, 20, and 30 mg/mL CS immediately after adult eclosion at 29°C throughout the experimental procedure. Our data demonstrated that the lifespan extension caused by CS was gender-dependent. As shown in Figure 1(a), there was no lifespan extension in male flies treated with different doses of CS. In contrast, CS resulted in a significant increase in the maximum lifespan of females in all CS-supplemented groups compared with the CS-free control group (Figure 1(b)). The maximum lifespan, shown by the 90th survival percentile, increased up to 11.4% with 10 mg/mL CS and 5.7% with both 20 and 30 mg/mL CS. We also found that the mean lifespan was significantly extended from 25.5 (control) to 29.4, 30.0, and 28.4 days upon treatment with 10, 20, and 30 mg/mL CS, respectively (all *P* < 0.001), and the maximum magnitude of increase in the mean lifespan was 1.18% at 20 mg/mL CS. Our results suggest that the addition of CS into the diet significantly extends lifespan. Because female flies were more responsive to CS, this sex was used for further experiments.

### 3.2. CS Supplementation Increases Resistance to Oxidative Stress and Starvation

**Figure 2:**
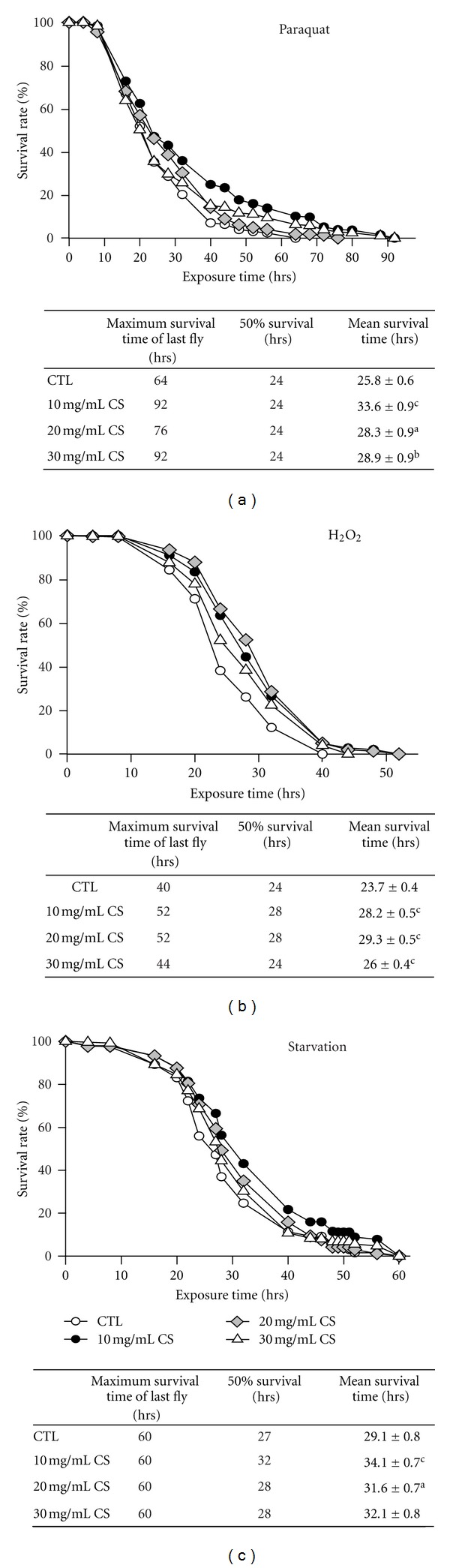
Effects of CS supplementation on resistance to oxidative stress and starvation. Female flies fed the control diet (CTL, open circle) and experimental diets supplemented with 10 (closed circle), 20 (open diamond), and 30 mg/mL (open triangle) CS at 29°C for 20 days. Oxidative stress tests with (a) paraquat, (b) H_2_O_2_, and (c) starvation treatments were then performed. Data represent the total survival time of the tested flies. Data are shown as the mean ± SEM and significantly different from the controls for each treatment at ^a^ *P* < 0.05, ^b^ *P* < 0.01, and ^c^ *P* < 0.001.

As shown in Figure 1(b), we found that the survival rates at 20 days were 77.5% for control females and 86.5, 94.0, and 85.3% for experimental groups supplemented with 10, 20, and 30 mg/mL CS, respectively. Because lifespan extension is often associated with enhanced resistance to oxidative stress during aging, flies pretreated with CS for 20 days were evaluated for their survival after challenges with paraquat and H_2_O_2_ which produce ROS to trigger cellular senescence and cell death. We found that CS protected females against paraquat toxicity and extended both the mean and maximum lifespan (Figure 2(a), *P* < 0.001, 0.05, and 0.01 at 10, 20, and 30 mg/mL, resp.). In the H_2_O_2_ challenge, flies fed CS, especially at the 20 mg/mL dose, survived longer than untreated controls (Figure 2(b), all *P* < 0.001), indicating that CS increased oxidative resistance against paraquat and H_2_O_2_ toxins. A similar trend showing increased resistance to starvation was also detected (Figure 2(c)). The mean survival time showed a significant increase in the 10 and 20 mg/mL CS-supplemented groups compared with the controls (*P* < 0.001 and 0.05, resp.) but not in the 30 mg/mL CS group. These results reveal that treatment with CS can improve resistance to some oxidative stress, suggesting that the lifespan extension induced by CS might be associated with an increase in antioxidative defense.

### 3.3. CS Increases Catalase Activity and Decreases LPO Levels

**Figure 3:**
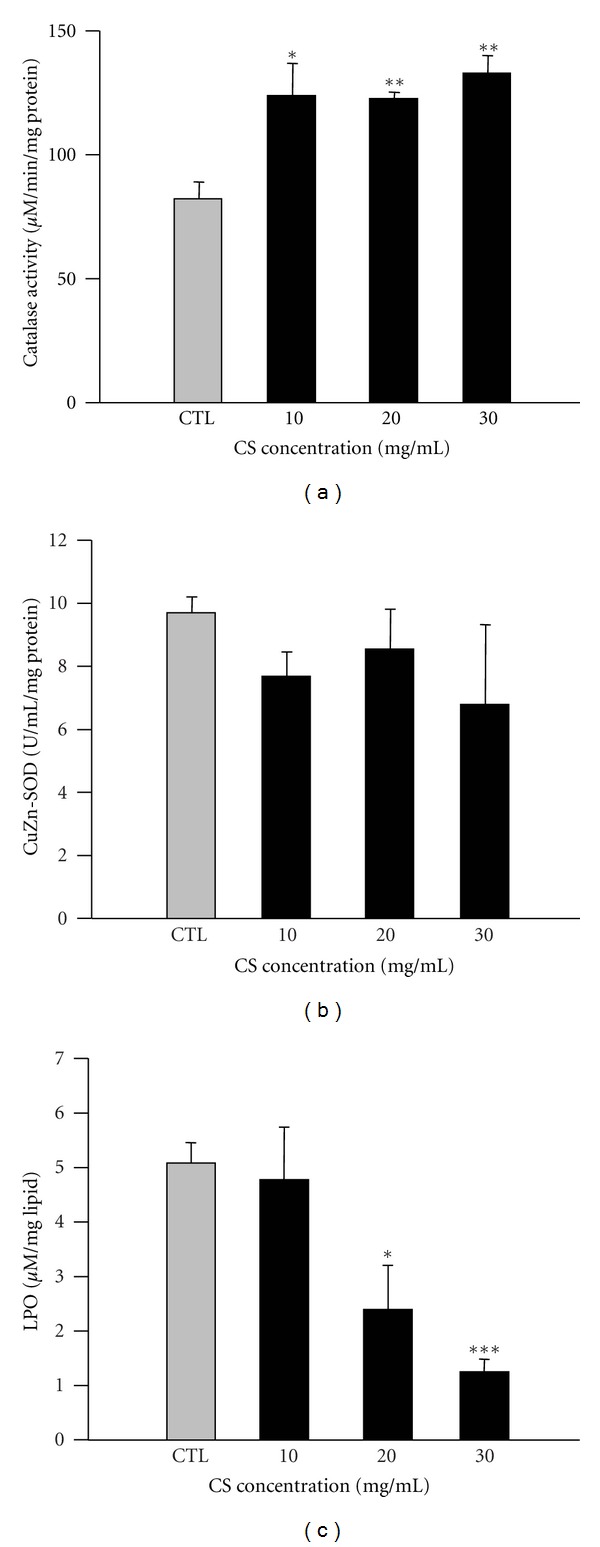
Effects of CS supplementation on the enzymatic activity of catalase, CuZn-SOD, and whole body LPO levels. Female flies were pretreated with the control diet (CTL, gray bar) and experimental diets containing 10, 20, and 30 mg/mL CS (black bar) at 29°C for 20 days. The enzymatic activity of (a) catalase, (b) CuZn-SOD, and (c) whole body LPO levels were determined. Data are expressed as the mean ± SEM. *P < 0.05, **P < 0.01, and ***P < 0.001 indicate significant differences compared with the control group.

To characterize the mechanism by which CS protects against oxidative stress, we evaluated the changes in free radical scavenging enzymes in CS-feeding flies, such as catalase and SOD. The data revealed increased catalase but not CuZn-SOD activity in the CS-supplemented groups compared with the controls (Figures 3(a) and 3(b)). As Figure 3(a) shows, for flies pretreated with CS for 20 days, catalase activity increased by nearly 49.4% for the 10 and 20 mg/mL CS diets (*P* < 0.05 and 0.01, resp.) and 61.8% for the 30 mg/mL CS diet (*P* < 0.01) compared with the control group. We also analyzed the levels of LPO, a process that refers to lipid oxidation caused by ROS, partly due to aging (Figure 3(c)). We observed a dose-dependent 53.0 and 75.4% reduction in total body LPO in the 20 and 30 mg/mL CS-treated flies versus the controls, respectively (*P* < 0.05 and 0.001, resp.). CS supplementation not only increased antioxidant activity through a change in catalase activity but also decreased LPO formation, suggesting that CS protects tissue from ROS attacks.

### 3.4. Effects of CS on Courtship Behavior

**Figure 4:**
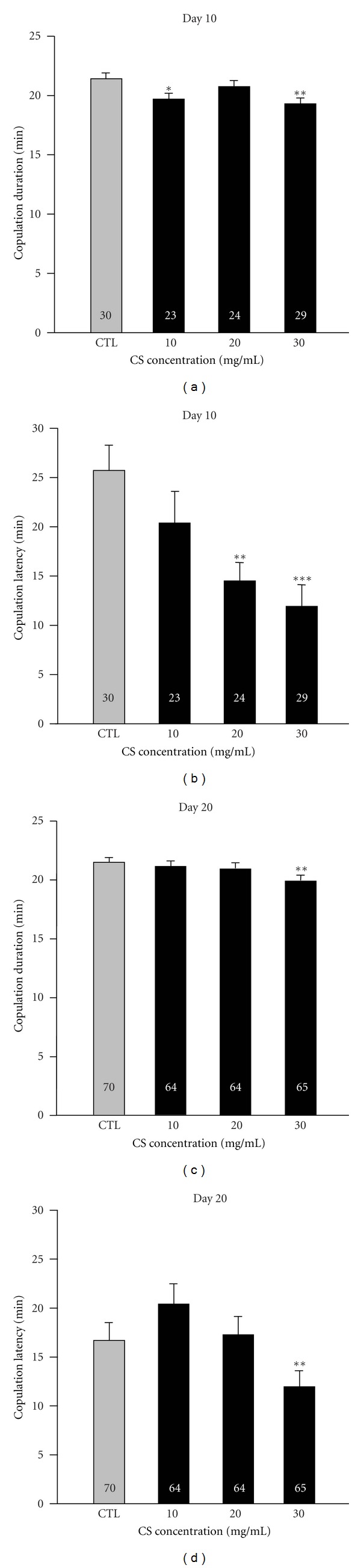
Effects of CS supplementation on courtship behavior. Female flies were pretreated with the control diet (CTL, gray bar) and experimental diets containing 10, 20, and 30 mg/mL CS (black bar) at 29°C for either 10 ((a), (b)) or 20 ((c), (d)) days. Copulation duration ((a), (c)) and copulation latency ((b), (d)) were determined. Data are expressed as the mean ± SEM. Sample size is shown in the corresponding bars for each treatment. *P < 0.05, **P < 0.01, and ***P < 0.001 indicate significant differences compared with the control groups.

Because CS supplementation in the diet induced a significant lifespan extension, we next asked whether CS had positive effects on fly courtship behavior. We recorded the times at which couples initiated and terminated copulation and calculated their copulation duration to analyze mating activity after CS supplementation. In our experiment, a virgin female fed varying doses of CS for 10 (young) or 20 days (aged) was paired with a 9-day-old control virgin male in a plastic chamber to determine their copulation duration (Figure 4). The copulation duration was significantly shortened by 9.8 and 7.4% in the experimental groups treated with 30 mg/mL CS for both young and old flies, respectively, compared with the CS-free controls (all *P* < 0.01) (Figures 4(a) and 4(c)). We also calculated the copulation latency, the times between which the couples were placed in the chamber and started to copulate. Females treated with 20 and 30 mg/mL CS for 10 days showed 43.7 and 53.7% shorter copulation latency than controls, respectively (Figure 4(b), *P* < 0.01 and 0.001, resp.), and this result also appeared in females with a 30 mg/mL CS diet for 20 days (28.5%, *P* < 0.01) (Figure 4(d)). Our observations indicate that females fed CS tended to engage in mating activity with males, and a higher CS dose caused females to shorten the mating process.

### 3.5. Effects of CS on Reproductive Capacity

**Figure 5:**
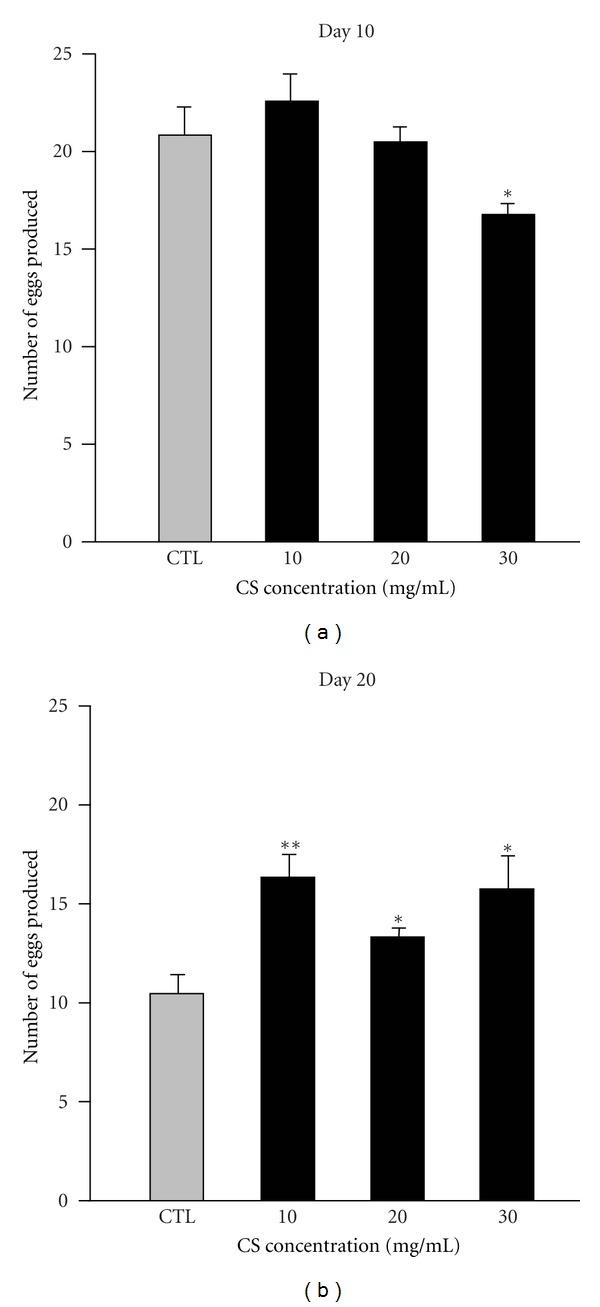
Effects of CS supplementation on reproductive capacity. Female flies pretreated with the control diet (CTL, gray bar) and experimental diets containing 10, 20, and 30 mg/mL CS (black bar) at 29°C for either 10 (a) or 20 (b) days were measured for fecundity. Data are expressed as the mean ± SEM. Units represent the mean daily egg production per female. At least five vials were used, and total fly numbers were *n* = 50–60 for each treatment group. *P < 0.05 and **P < 0.01 indicate significant differences compared with the control groups.

Because the theory of aging indicates that the aging process negatively affects reproduction, we tested whether CS impacted reproductive fitness by analyzing the egg-laying ability of adult female flies. We found significant increases in the daily number of eggs laid per female by 55.8, 26.9, and 50.0% in aged flies treated with 10, 20, and 30 mg/mL CS compared with the CS-free controls, respectively (Figure 5(b), *P* < 0.01, 0.05, and 0.05, resp.), but for young flies, the higher dose of CS (30 mg/mL) caused a moderate decrease in egg-laying ability (19.5%, *P* < 0.05) (Figure 5(a)), suggesting that CS could facilitate increased egg production in aged but not young flies.

### 3.6. CS Delays Age-Related Learning Impairment

**Figure 6:**
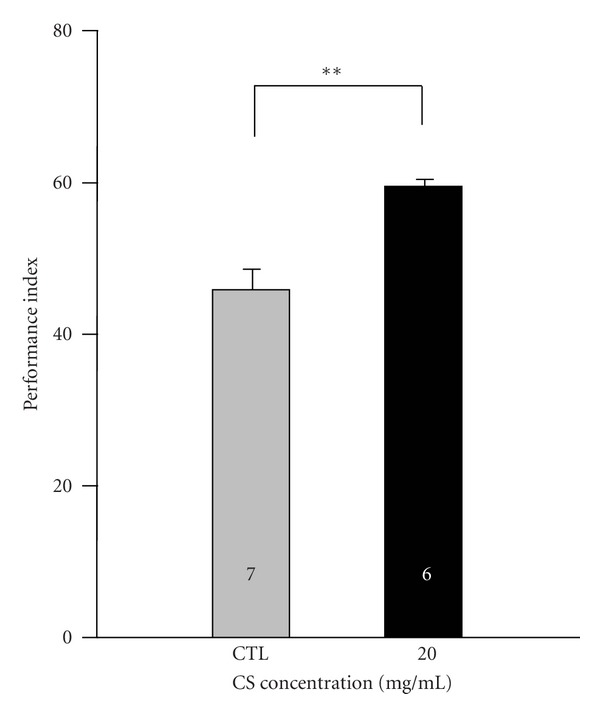
Effects of CS supplementation on cognitive behavior. Female flies were pretreated with the control diet (CTL, gray bar) and experimental diets containing 20 mg/mL CS (black bar) at 29°C for 20 days. Data are expressed as the mean ± SEM. Sample size is shown in the corresponding bars for each group. **P = 0.0011 indicates the significant difference compared with the control group.

While feeding CS to increase fly's longevity and oxidative resistance has been identified in our present study, the effects of these longevity-promoting factors on cognitive behavior are unknown. We attempted to ascertain whether CS administration could also improve learning behavior in aged flies. Because supplementation of CS at 20 mg/mL shows a significant effect on survival rate (Figure 1(b)), flies exposed to standard food with or without 20 mg/mL CS for 20 days tend to mimic aging, and a single cycle of olfactory associative training was performed to evaluate their learning ability (Figure 6). We found that flies supplemented with CS had a higher performance index of learning (59.7 ± 1.0) than control flies (46.0 ± 2.8) (*P* = 0.0011), suggesting that CS has a beneficial effect on the maintenance of learning ability with age.

## 4. Discussion

This study shows for the first time that adult-onset CS supplementation in flies enhances longevity and increases resistance to paraquat, H_2_O_2_, and starvation stress challenges. We also found that CS plays crucial roles in modulating mating readiness, reproductive capacity, and cognitive behavior. These results indicate that CS delays age-related syndromes during the aging process.

We initially investigated whether CS could prolong lifespan in both sexes of flies; unexpectedly, the lifespan extension induced by CS supplementation was only observed in females. The mean lifespan increased in females for all three tested doses, with the largest increase observed at 20 mg/mL CS (Figure 1(b)), suggesting that females flies are more responsive to CS supplementation. A similar gender effect on lifespan has been reported for other nutritional and genetic interventions. Flies fed with resveratrol, curcumin, or acai palm fruit, as well as those overexpressing dFOXO in adult fat body, lived longer, particularly in females [9, 26–28]. As studies in mammals about the target of rapamycin (TOR) signaling pathway that modulate longevity report, mice lacking the ribosomal S6 protein kinase 1 (S6K1) or fed with rapamycin showed an extended lifespan more prominently in females [29, 30]. However, the reasons for this gender-specific influence are unclear and merit further study.

The underlying mechanisms by which CS extends the mean and maximum lifespan of flies remain obscure. Because aging is correlated with oxidative damage caused by ROS attacks, it is speculated that elevating oxidative resistance could yield a prolongevity effect [31, 32]. We tested this hypothesis, and our observations indicated that flies pretreated with CS exert a higher protective effect against oxidant insults from paraquat and H_2_O_2_, which generate free radical superoxide anions and hydroxyl radicals, respectively. We also examined changes in antioxidative defense systems, especially catalase and CuZn-SOD. CS altered catalase enzyme activity but made no changes to CuZn-SOD, in contrast with what is observed for green tea extract, *Rhodiola rosea*, and *Rosa damascene*, which also extend fly lifespan [6, 33, 34]. Green tea extract elevated both catalase and SOD activity, but *R. rosea* and *R. damascene* failed to change these antioxidant enzymes. Our data suggest that CS may work via catalase pathway to protect flies against elevated oxidative stress with age.

Because we found that the administration of CS in adults could decrease fly mortality and improve tolerance to oxidant stress, we examined whether CS could spawn other benefits, especially in courtship behavior and reproductive capacity, which are both negatively correlated with age [2]. Copulation duration was significantly reduced at a dose of 30 mg/mL CS in both young and aged flies, suggesting that a higher dose of CS might cause the effect of shortened copulation duration. We also observed that the copulation duration was similar for both young and old control wild-type couples, a result seemingly different from previous studies. In the *Drosophila* species *D. pavani* and *D. gaucha*, copulation duration varies with age, with older female flies showing longer copulation duration than younger flies [35]. In *D. melanogaster*, the copulation duration is also correlated with age. Beaver et al. found that 4-day-old males copulated for shorter durations than 2-day-old males [36]. Compared with our results, this variation might be due to the different sexes, species, and ages of flies we tested. According to the copulation latency analysis, we observed older females mating faster than young flies treated with control food, and females supplemented with CS exhibited higher mating readiness in a dose-dependent manner. These results are consistent with the clinical use of CS for improving sexual behavior in humans.

Prior studies have highlighted the negative correlation between lifespan extension and fecundity. Flies that undergo downregulation of the insulin signaling pathway, with DR conditioning, or supplemented with *R. rosea* can live longer but show impaired fecundity [33, 37, 38], while females overexpressing dFOXO in the head fat body or supplemented with curcumin showed lifespan extension and normal fecundity [9, 39]. Wood et al. reported that resveratrol resulted in female flies having an extended lifespan and increased egg production at a young age [40]. In contrast to the above studies, our data showed that CS supplementation is sufficient to augment both the lifespan and fecundity of aged female flies. CS supplementation significantly increased fecundity in aged flies at all three doses. No effect on fecundity was observed in young flies with 10 or 20 mg/mL CS, but the higher dose of CS (30 mg/mL) moderately decreased egg-laying ability. Taken together, the higher fecundity in aged females may not be directly linked with the copulation phenotype but instead with the pharmacological properties of CS, suggesting that CS not only mediates lifespan extension but also enhances female courtship behavior and reproductive function during the aging process.

Functional changes in synapses that contribute to altered neuronal plasticity and cognitive decline have a well-described association with age in mammals [41, 42]. In our study, the reason for the CS-related suppression of age-related learning impairment is unclear. CS might play a role in regulation of neurotransmitter transport. Based on screening of cells expressing various neurotransmitter transporters, CS extracts raised dopamine and norepinephrine uptakes but showed decreases in GABA and serotonin [43]. The regulation of synaptic transmission activity by transmitter systems is frequently correlated with cognitive performance. Serotonin and dopamine neurotransmitters are strongly associated with learning and memory in brain areas such as the prefrontal cortex, hippocampus, and corpus striatum [44, 45], while GABA is the principle inhibitory neurotransmitter that plays a regulatory role in protecting neurons from hyperexcitation in neuronal circuits and also modulates spatial and emotional behavior in rodents [46]. The classical neurotransmitter glutamate and its ionotropic receptors (AMPA and NMDA subtypes) are intimately involved in memory formation [47]; however, whether this signaling pathway is involved in CS, alleviating learning impairment in aged flies, remains unknown.

Another mechanism for the retention of learning ability apparent in CS-fed aged flies may be correlated with enhanced antioxidant defense systems. A previous study indicated that CS has neuroprotective effects against staurosporine-induced apoptosis in human neuroblastoma cells [48]. While staurosporine-induced neuronal injury is associated with greater ROS production, this result suggests that CS has the potential to protect cells from damages caused by oxidative stress, a risk factor in the aging process. Some reports have demonstrated that enhancement of antioxidant capability is able to suppress cognitive impairment in aged animals. Liu et al. showed that chronically administering two compounds to scavenge ROS almost totally reversed learning deficits in the aged mice [49]. Supplementation of extra virgin olive oil increased brain glutathione levels and improved age-related learning and memory deficits in a dementia animal model of SAMP8 mice [50]. When receiving a long-term diet with blueberry extracts, kainate-treated rats exhibited less CA1 pyramidal neuron loss and better learning performance, suggesting a protective role for blueberries against neuronal excitotoxicity and oxidative stress [51]. In the DR conditioning, SAMP8 mice generated less ROS and increased some neurotransmitter levels, including acetylcholine, dopamine, and norepinephrine [52]. DR has also been suggested to help retain better cognitive ability in old rats [53]. Here, we report that CS supplementation can extend fly lifespan and also increases lifespan under varied oxidative stress, suggesting that CS might have an antioxidant effect that suppresses age-related learning deficits during the aging process.

## 5. Conclusion

The present results provide clues as to how CS could alleviate age-related syndromes, especially in terms of brain function, courtship behavior, and reproductive capacity, when CS is provided at a younger age. Our study provides some basic evidence supporting clinical treatment of replenishing the vital essence in traditional Chinese medicine and might provide valuable insight for developing new drugs or food additives to delay aging and the progression of age-related diseases.
